# ELISA versus PCR for diagnosis of chronic Chagas disease: systematic review and meta-analysis

**DOI:** 10.1186/1471-2334-10-337

**Published:** 2010-11-25

**Authors:** Pedro EAA Brasil, Liane De Castro, Alejandro M Hasslocher-Moreno, Luiz HC Sangenis, José U Braga

**Affiliations:** 1 Instituto de Pesquisa Clínica Evandro Chagas - Fundação Oswaldo Cruz, Rio de Janeiro/RJ, Brazil; 2 Instituto de Medicina Social - Universidade do Estado do Rio de Janeiro, Rio de Janeiro/RJ, Brazil; 3 Centro de Referência Professor Hélio Fraga - Escola Nacional de Saúde Pública - Fundação Oswaldo Cruz, Rio de Janeiro/RJ, Brazil

## Abstract

**Background:**

Most current guidelines recommend two serological tests to diagnose chronic Chagas disease. When serological tests are persistently inconclusive, some guidelines recommend molecular tests. The aim of this investigation was to review chronic Chagas disease diagnosis literature and to summarize results of ELISA and PCR performance.

**Methods:**

A systematic review was conducted searching remote databases (MEDLINE, LILACS, EMBASE, SCOPUS and ISIWeb) and full texts bibliography for relevant abstracts. In addition, manufacturers of commercial tests were contacted. Original investigations were eligible if they estimated sensitivity and specificity, or reliability -or if their calculation was possible - of ELISA or PCR tests, for chronic Chagas disease.

**Results:**

Heterogeneity was high within each test (ELISA and PCR) and threshold effect was detected only in a particular subgroup. Reference standard blinding partially explained heterogeneity in ELISA studies, and pooled sensitivity and specificity were 97.7% [96.7%-98.5%] and 96.3% [94.6%-97.6%] respectively. Commercial ELISA with recombinant antigens studied in phase three investigations partially explained heterogeneity, and pooled sensitivity and specificity were 99.3% [97.9%-99.9%] and 97.5% [88.5%-99.5%] respectively. ELISA's reliability was seldom studied but was considered acceptable. PCR heterogeneity was not explained, but a threshold effect was detected in three groups created by using guanidine and boiling the sample before DNA extraction. PCR sensitivity is likely to be between 50% and 90%, while its specificity is close to 100%. PCR reliability was never studied.

**Conclusions:**

Both conventional and recombinant based ELISA give useful information, however there are commercial tests without technical reports and therefore were not included in this review. Physicians need to have access to technical reports to understand if these serological tests are similar to those included in this review and therefore correctly order and interpret test results. Currently, PCR should not be used in clinical practice for chronic Chagas disease diagnosis and there is no PCR test commercially available for this purpose. Tests limitations and directions for future research are discussed.

## Background

Chagas disease is an infection, in which the necessary cause is a parasite called *Trypanosoma cruzi*. This disease is endemic in Latin American countries and approximately 15 million people are estimated to be infected [1]. With progressive control of vector borne transmission in the majority of Latin American countries,[1] much attention has been given to the possibility of Chagas disease spread outside Latin America through blood donation and/or organ transplants, due to the increasing migration of Latin Americans around the world [2]. Case reports of Chagas disease from countries in which this infection is not typically endemic, such as France[3], Canada[4-6], Switzerland[7], Denmark[8], Germany[9], USA[10-12], and Spain[13,14] indicate that in the appropriate clinical situation, Chagas disease should be considered as differential diagnosis not only in Latin Americans, but also in individuals who are not from Latin America.

One significant difficulty in diagnosing Chagas disease is that most patients have no symptoms in acute or chronic phase [2,15,16]. Another difficulty in diagnosis is that, unlike most infectious diseases, the direct or parasitological tests for Chagas disease (thick or thin smear, microhematocrit, hemocultures or xenodiagnosis) have unacceptably low sensitivity in the chronic phase, ranging from 50% to 70%,[17] and are not recommended [15-19]. Thus, the diagnosis relies almost solely on serological tests.

Screening blood donors for Chagas disease is of much concern in all Latin American countries. Although the World Health Organization (WHO) expert committee and some guidelines recommend a single enzyme linked immunosorbent assay (ELISA) test to screen blood donors,[16,18,19] in some countries, such as Brazil[15], there is a more restrictive regulation, recommending two simultaneous (in parallel) tests of different techniques. Due to potential transmission of Chagas disease through blood transfusion, the United States of America, Spain and other non Latin American countries also screen blood donors for Chagas disease [20,21].

Currently, Pan-American Health Organization (PAHO) recommendations[16] and other guidelines[2,15,17,18] advise the use of two different serological techniques for chronic Chagas disease diagnosis, one of the techniques being ELISA. The basis of this recommendation is not clear, although some authors claim it to be due to poor concordance between ELISA and other serological tests,[22-25] and others claim it is due to limited specificity [2]. It is known that ELISA tests, as most tests used for screening purposes, may occasionally lead to false positive results, which must be confirmed later by other assays.

A pitfall of conventional ELISA is the possibility of cross-reaction with antibodies from patients infected with *Leishmania sp*. or *T. rangeli *[26-28]. This is a difficult problem to solve where these infections share endemicity with Chagas disease. In an attempt to overcome these limitations, efforts were made to develop ELISA with recombinant antigens (ELISA-rec) and polymerase chain reaction (PCR) tests for Chagas disease.

Currently, PCR test may be recommended depending on the situation and guideline considered. PCR test is recommended for chronic Chagas disease diagnosis only when serological tests are inconclusive by the Brazilian and Chilean consensus; [15,17] may be recommended only as confirmatory test after screening of blood donors according to El Salvador's guideline;[16] it is recommended only for acute or congenital infection diagnosis or therapy follow-up after acute infection diagnosis according to WHO expert committee[19] and a north American review;[2] and according to Spanish consensus a patient is considered with Chagas disease either with two positive serological test or PCR (or other parasitological) positive test when chronic disease is suspected [18]. Thus, the use of PCR tests for chronic Chagas disease diagnosis is controversial.

The aims of this investigation were summarize sensitivity, specificity and diagnostic odds ratio (DOR) for ELISA, ELISA-rec and PCR; evaluate the heterogeneity within this literature for chronic Chagas disease; and compare the overall accuracy of these three tests.

## Methods

This investigation was designed as a systematic review and meta-analysis.

### Search strategies

Three bibliographic methods were used to identify potential abstracts or investigations: remote search in electronic databases; bibliographic citations from included and excluded full text retrieved from other search methods; email contact with manufacturers that had any device or diagnostic kit concerning Chagas disease registered at ANVISA (Agência Nacional de Vigilância Sanitária - or the Brazilian National Agency of Sanitary Surveillance) through October 2007. No hand search or contacts with experts were made.

Electronic searches were executed in five different databases on June 6^th^, 2007: PubMed/Medline; SCOPUS; LILACS; ISIWeb/Web of Science; and EMBASE. The following strategy was developed in PubMed/Medline using clinical queries for diagnostic studies maximizing the sensitivity of the search: ("Chagas Disease"[MeSH] OR "Trypanosoma cruzi"[MeSH]) AND (ELISA OR (enzyme AND linked AND assay) OR PCR OR (polymerase AND chain AND reaction))) AND (sensitiv*[Title/Abstract] OR sensitivity and specificity[MeSH Terms] OR diagnos*[Title/Abstract] OR diagnosis[MeSH:noexp] OR diagnostic*[MeSH:noexp] OR diagnosis, differential[MeSH:noexp] OR diagnosis[Subheading:noexp] OR "Reproducibility of Results"[Mesh] OR reliability OR reproducibility). This strategy was adapted to the other four bases and they were all updated on April 20^th^, 2009.

In December 2007, the ANVISA website http://www7.anvisa.gov.br/datavisa/Consulta_Produto_correlato/consulta_correlato.asp was accessed to check for medical products, devices or kits related to Chagas disease. At the time, there were 52 registries, and only seven were active. Since technical reports used to register these products are not available at ANVISA website, emails were sent to manufacturers or their legal distributors requesting the technical report, monographs, non-published literature or a reference of a published report related to their product.

### Inclusion/exclusion criteria

The abstracts were eligible for full text evaluation if their aims were: estimate sensitivity or specificity of one or more ELISA or PCR for chronic Chagas disease; estimate accuracy of an ELISA or PCR for chronic Chagas disease; to test a new ELISA or PCR for chronic Chagas disease; to estimate any validity measure for ELISA or PCR for chronic Chagas disease such as likelihood ratios, accuracy, error rate, DOR, area under the ROC (receiver operating characteristics) curve or predictive values; to estimate intra-test variability (reliability) of a PCR or ELISA for chronic Chagas disease. Abstracts with unclear objectives, but which partially met any of the criteria above, or with unclear objectives and had any of the validity measure (as described above) as a result, were also included.

Abstracts indicating that the investigation was neither conducted with human volunteers nor with samples from human beings, or indicating that the tests were studied in a verification of cure scenario were not included. Investigations concerning exclusively acute infection or newborns, or with mixed data from acute and chronically infected patients were excluded.

After full text retrieval the following criteria were applied for quality evaluation and data extraction: (a) investigations should be original (no reviews, editorials or letters); (b) should be quantitative investigations; (c) every investigation must have two samples (one representing those with chronic Chagas disease and one representing those without chronic Chagas disease); (d) must have results with enough data to allow extraction (or calculation) of true positives, false negatives, false positives and true negatives of each test. Only texts published after 1980 were included. Although only abstracts in English, Spanish or Portuguese were accepted, no language restriction was applied to full text evaluation.

All diagnostic investigation phases were accepted. Investigations were classified into these phases according to Haynes [29]. Briefly, studies were classified as (1) phase 1 if they compared results distributions of those known to have disease with those known not to have disease, usually in small samples in which selection was done by convenience or by previous knowledge of Chagas disease status; (2) as phase 2, if they estimated sensitivity and specificity (or predictive values) from study designed as case-control, that could also use the same data from a phase one study; (3) as phase 3, those studies designed as cross-sectional with consecutive or random selection of the volunteers, where the main inclusion criteria is based on the suspected chronic Chagas disease.

### Index text and reference standard

The target condition of interest was chronic Chagas disease. Classifying Chagas disease according to infection time (in acute and chronic) may be challenging. Usually chronic Chagas disease means adults with long term infection (ten years or longer), but the term may also be applied to children, primarily with the indeterminate form of the disease, with no history of acute fever detected in the past two months. Original investigations with children were also included if the authors did not define their infection as either acute or chronic, and there was no report of symptoms or signs compatible with acute phase. Studies with children with one year old or less were considered as acute infection studies.

All reference standards used by the authors of the original investigations were accepted. This was based on the rationale that for a long time, there was no 'gold standard' for Chagas disease diagnosis accepted among all experts. However, two or more simultaneous serological tests (with at least two different techniques) were considered appropriate in the review quality assessment. The index tests (tests under evaluation) of interest of this research were ELISA, ELISA-rec and PCR.

From 1980 to 2010, ELISA technology has been widely accepted and used due its automation and ease of use (most of the tests are semi-automated). Its results are less operator dependent, and are more readily available. The basis of the test is to detect patient antibodies against *T. cruzi *antigens. This serological test is a reaction in which an enzyme gives a colorful result if the serum sample has the target anti-body, and it is recorded in optical densities.

The most interesting variation is the ELISA-rec. This variation does not use antigens made from lysates of whole parasites. Instead, its antigens (peptides) are constructed with recombinant technology. All variations were included and discriminated.

PCR technique for Chagas disease is considered a parasitological test such as thick or thin smear, xenodiagnosis or hemocultures, because the test relies on amplification of DNA (Deoxyribonucleic acid) target sequences. The test is based on the detection of *T. cruzi *DNA sequences in patients' blood samples.

There are different PCR techniques (qualitative methods) for detecting *T. cruzi *DNA in patients' blood samples are: "Hot-Start PCR" (a modification of conventional PCR that should reduce nonspecific amplification during the initial setup stages); "Nested-PCR" (two step amplification often used for very low amount of DNA targets); "Southern Blot" or "PCR and hybridization" (a procedure used to verify the presence or absence of a specific nucleotide sequence in the DNA from patients' blood samples by a labeled hybridization probe).

In some analysis, it is important to detect the amounts of pathogens, so as to indicate the disease severity or to monitor therapy outcome of infected patients. The Real-Time PCR is based on the polymerase chain reaction technology, used to amplify and simultaneously quantify a targeted DNA molecule.

There are primers for two main target regions of *T. cruzi *DNA amplification: nuclear satellite DNA (ns-DNA) - a family of highly repetitive nuclear DNA sequences named E13, that is distributed over most of the parasite chromosomes;[30] and Kinetoplast DNA (K-DNA) - part of the usual mitochondrial DNA found in trypanosomes [31]. All these variations were included and discriminated.

### Review process

Research forms were designed and piloted for the purpose of this review. Four blinded reviewers evaluated abstracts and full text. One reviewer read and classified all abstracts and eligible full text, and each one of the remaining three reviewers reviewed and classified approximately one third of the abstracts. Disagreement among reviewers was resolved in consensus meetings and tended to be inclusive if disagreement was persistent. In a similar way, two blinded reviewers classified and extracted each full and disagreement was solved in a consensus meeting.

### Methodological quality assessment

The methodological quality of each included investigation was also evaluated in a blinded fashion with QUADAS tool (QUality Assessment of Diagnostic Accuracy Studies) [32,33]. A consensus meeting was conducted to confirm agreement and to resolve disagreement between reviewers about this issue.

QUADAS is a checklist to help readers assess key issues about quality of conducting and reporting of diagnostic test research, which will help in results interpretation. Its interpretation is rather qualitative, making comparison of quality only possible through individual items. Overall scores or grades may not help quality interpretation and are not recommended [34-36].

### Data analysis plan

**Figure 1:**
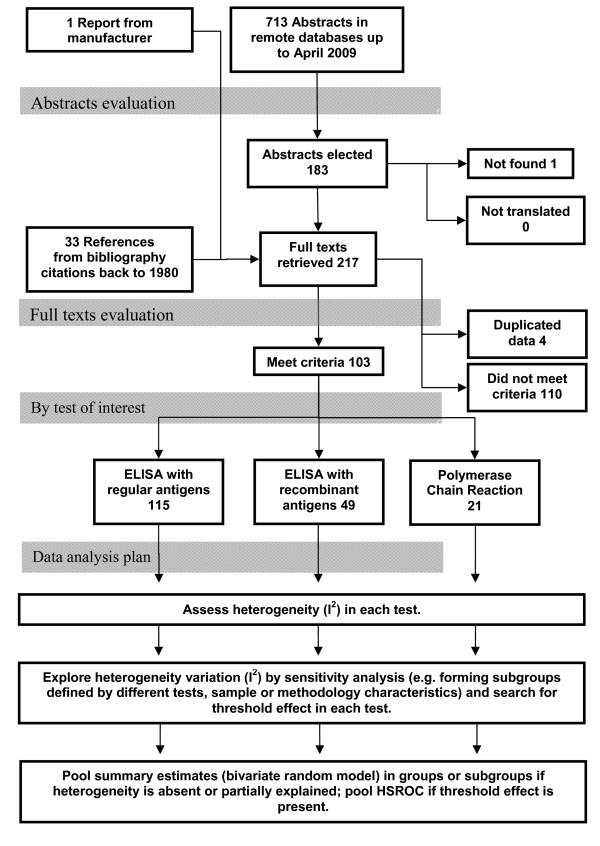
**Flowchart of abstracts and full texts evaluation and data analysis plan**. Several full texts evaluated two or more tests; therefore the sum of tests with data extracted is not equal to the total of full text included.

The statistical analysis was based on the following steps (figure 1): (1) qualitative description of findings; (2) search for the presence of heterogeneity and a threshold effect; (3) exploring possible explanations for heterogeneity by sensitivity analysis; (4) statistical pooling. TeleForm^® ^was used for data entry and analysis was carried out with R-project software[37], with packages meta[38] and DiagMeta [39].

One must understand heterogeneity as a greater variation of sensitivity, specificity or DOR between the included studies than is compatible with the play of chance. This statistical heterogeneity should represent other sources of heterogeneity such as: clinical characteristics, tests characteristics or research design characteristics. It was decided a priory that sources of heterogeneity investigation would be conducted by sensitivity analysis, in other words, changing inclusion and exclusion criteria for sub-group analysis, including and excluding one by one of the information available (shown in Additional file 1, Additional file 2 and Additional file 3 and others) such as: tests characteristics (such as different antigens, different methods for estimating the cut-off, different DNA region targets etc); sample or populations characteristics (such as age range, sex, location; if studied in blood donors); and design/report characteristics, including QUADAS items (such as the reference standard, blinding, if submitted to ethics evaluation etc.). Study phase and whether the test was commercially available were of particular interest.

Heterogeneity was explored with I^2 ^estimate [40]. I^2 ^measures up to 25% were considered low evidence of heterogeneity, between 25% and 50% with moderate heterogeneity, and 50% or higher with high heterogeneity. The I^2 ^was estimated in the sensitivity, specificity and DOR measures.

Threshold effect was investigated as a source of heterogeneity and to check if pooling summary ROC was appropriate. It was defined as a positive correlation among true positive rate (TPR) and false positive rate (FPR) - or a negative correlation among sensitivity and specificity - and was explored in a HSROC model (Hierarchical Summary Receiver Operator Characteristics) fitted by Maximum Likelihood (ML) and Monte Carlo Markov Chain (MCMC) methods. If there was a moderate to high heterogeneity, correlation was explored, and if positive, a HSROC was estimated by principal component of positive MCMC estimations[39] and plotted, if negative or zero summary estimates were pooled without HSROC [39].

Summaries estimates - Sensitivity (or True Positive Rate) and 1- Specificity (or False Positive Rate) - were pooled using the random bivariate model, with Laplace method for Maximum Likelihood (ML) and Monte Carlo Markov Chain (MCMC) method [39]. Summary DOR was estimated as the ratio of positive likelihood ratio over negative likelihood ratio with DerSirmonian & Laird random effect using the inverse variance technique for sample size weight.

## Results

### Search results

From all five remote databases, 1349 abstracts were retrieved. Following the removal of replicates, 713 were found and evaluated (figure 1). After abstracts evaluation only 183 were elected for retrieval of the full text.

Based on the strategy of email or telephone contact with manufacturers or distributors, 15 out of 57 ANVISA registries were not used, either because some were clearly not about ELISA tests or, in four cases, because no email or phone contact could be found either at ANVISA registries, or in the World Wide Web. From this strategy only four returned the request.

Abbott-Brazil returned the operational manual for 'Chagas Test ELISA III', which was not considered a report to be evaluated although there was some sensitivity and specificity data. Lemos Laboratory (Argentina) sent a reference published in 1998 containing data from Polychaco/Biozima-Chagas, which was subsequently found in the remote databases search.

Ebram Laboratory returned the email stating that they did not have any technical report at the time, and the only validation process was executed by CGLAB (N° 03/06) (Coordenação Geral de Laboratórios de Saúde Pública - or General Coordination of Public Health Laboratories, which is a coordination in the Brazilian Epidemiologic Surveillance Department). http://portal.saude.gov.br/portal/arquivos/pdf/nota_kit_chagas.pdf However, this note could not be considered for evaluation because there was no data for extraction and no information to evaluate the quality of the investigation.

Orgenics sent a non-published report about the 'ImmunoComb II Chagas Ab kit'. Although described as a conventional ELISA, this test is formatted as a strip or rapid test. Even described as a multicenter evaluation, each center was considered a single report due to several protocol differences in each center. This report was the only one included from the manufacturer email contact search strategy.

Another concerning issue is that five out of the twelve commercial kits tested by CGLAB in 2006 were not found in the ANVISA list of registered medical products captured in 2007, nor were their reports found through other search strategies.

Thirty three references, that were not captured by remote databases search, were identified throughout bibliography of 2 narrative reviews[41,42] and other original investigations. Therefore, there were 217 full texts elected for assessment. (figure 1)

After applying inclusion/exclusion criteria, 114 texts were excluded, 4 of them were discarded due duplicated data in different reports by the same author - leaving 103 texts for data extraction. Several reports had data from two or more tests simultaneously; therefore, there were 115 regular ELISA reports[22,25,43-105] (Additional file 1), 49 ELISA-rec reports [22,23,61,76,79,80,85,90,92,97,98,103,106-116] (Additional file 2) and 21 PCR reports[26,100,117-130] (Additional file 3), generating 185 tests results to analyze (figure 1).

### Methodological quality of included studies

Quality of included original investigations was assessed with QUADAS (Additional file 4). Only 8 (4.3%) investigations were considered to have clear inclusion criteria. Sixty three investigations (33.9%) were classified as using a reference standard likely to correctly classify Chagas disease. In 93 investigations (50.0%) it was clear that the whole sample received verification using the reference standard. In 106 investigations, it was clear that patients received the reference standard regardless of index text result. In 132 (71.0%) investigations, it was clear that the reference standard was independent from the index test. In 124 (66.7%) and 33 (17.7%) investigations, the index test and the reference standard respectively were clearly described. In 22 (11.8%) studies, index test or reference standard blinding was cited, but only in 18 (9.7%) were both included. In 20 (10.8%) studies, inconclusive index test results appeared to be omitted and in 19 (10.2%) withdrawals were not explained.

### Findings

There was a lot of information not available about clinical characteristics and sample description of the 185 tests from original investigations. (Additional file 1, Additional file 2 and Additional file 3) Only 20 (10.8%) specified the recruitment period, 31 (16.7%) specified the proportion of children in its sample, 28 (15.1%) specified the sex distribution, 32 (17.2%) specified age range, in 69 (37.1%) it was not possible to determine if there were blood donors in the sample, in 163 (87.6%) and 167 (89.8) it was not possible to determine if people living in rural or urban area respectively were included in the sample, in 26 (14.0%) the sample was composed exclusively of blood donors, in only 21 (11.3%) there was some information about clinical characteristics such as cardiac or digestive involvement, or disease severity, and in 31 (16.7%) submission to review board and ethics evaluation was reported

Eighty one investigations were conducted in Brazil, 24 in Argentina and the remaining were conducted in several other countries in Latin America, USA or Spain. In 9 (4.8%), it was not possible to determine where the protocol was conducted or from where included patients/samples were.

Concerning only the ELISA group, two tests were based on strip (rapid test) technology, in 76 (46.1%) tests it was not specified how the cut-off was estimated and only in 11 (6.7%) the cut-off estimation somehow considered the distribution of results of those with the target disease. In 143 (86.7%), it was not specified if there was an inconclusive range; in 110 tests (66.7%), the value of the cut-off was not specified.

In regular ELISA group, the strain used to extract antigens for the test was not specified in 68 (58.2%), and in all the commercial tests this information was not available; in 59 (50.7%) tests the *T. cruzi *life cycle form used as sources of antigens was not specified, and in 65 (56.0%) antigen purification was not specified or not clear in the report.

From the 186 tests evaluated, only 60 (32.4%) were specified to be commercially available. From these, 3 (5.0%) were classified as phase 1, 49 (81.7%) were classified as phase two, and only 8 (13.3%) were classified as phase 3.

Concerning the PCR group (Additional file 3), one protocol specified 3 blood samples collections from the same patients, four did not inform how many samples were collected and the remaining collected one blood sample; blood volume collected in each sample ranged from 1 ml to 15 ml; six did not inform storage condition and none informed time gap between blood collection and DNA extraction; ten studies added guanidine and boiled the blood samples before DNA extraction, five studies added guanidine and did not boiled the blood samples, five studies did not add guanidine neither boiled before DNA extraction, and in one study guanidine addition and boiling information was not available; extracted volume from each sample ranged from 100 μl to 500 μl and two studies did not inform extraction volume; eighteen studies used phenol-chloroform, two used commercial kits for DNA extraction and one did not inform DNA extraction procedure; eleven did not inform inhibition control procedure; eight did not inform contamination control procedure; only three studies informed analytical sensitivity; fourteen studies used primers aiming K-DNA and four studies used primers aiming ns-DNA; sixteen studies used protocols of regular PCR, three studies used hybridization PCR, one used a nested PCR and one used a real-time and nested PCR; annealing temperature ranged from 55°C to 65°C. All PCR tests were classified as in-house and 7 different combinations of primers were used. (Additional file 5)

Heterogeneity within each of the three tests (ELISA, ELISA-rec and PCR) was very high. (Additional file 6) In none of the groups explored, heterogeneity was fully explained. Also, threshold effect within the three tests was absent, which makes HSROC estimates or comparison of curves between them not appropriate.

**Figure 2:**
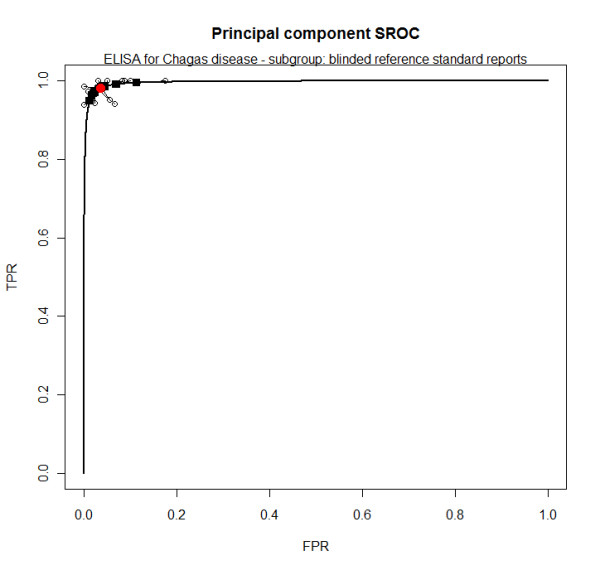
**Regular ELISA for Chagas disease summary ROC for tests with blinded reference standard evaluation**. Dots - crude estimates; filled black squares - shrunken estimates from bivariate model; filled red circle - bivariate model summary estimate; black solid line - SROC curve.

In the ELISA group, those tests that registered a blinded evaluation of the reference standard (QUADAS 11 = Yes) had moderate heterogeneity in sensitivity, moderate to high heterogeneity in specificity and little evidence of heterogeneity in DOR. A threshold effect was detected (Additional file 7) in this group and HSROC was considered appropriate. (figure 2) Within this subgroup, summary sensitivity and specificity were 97.7% and 96.3%, respectively. (Additional file 7)

**Figure 3:**
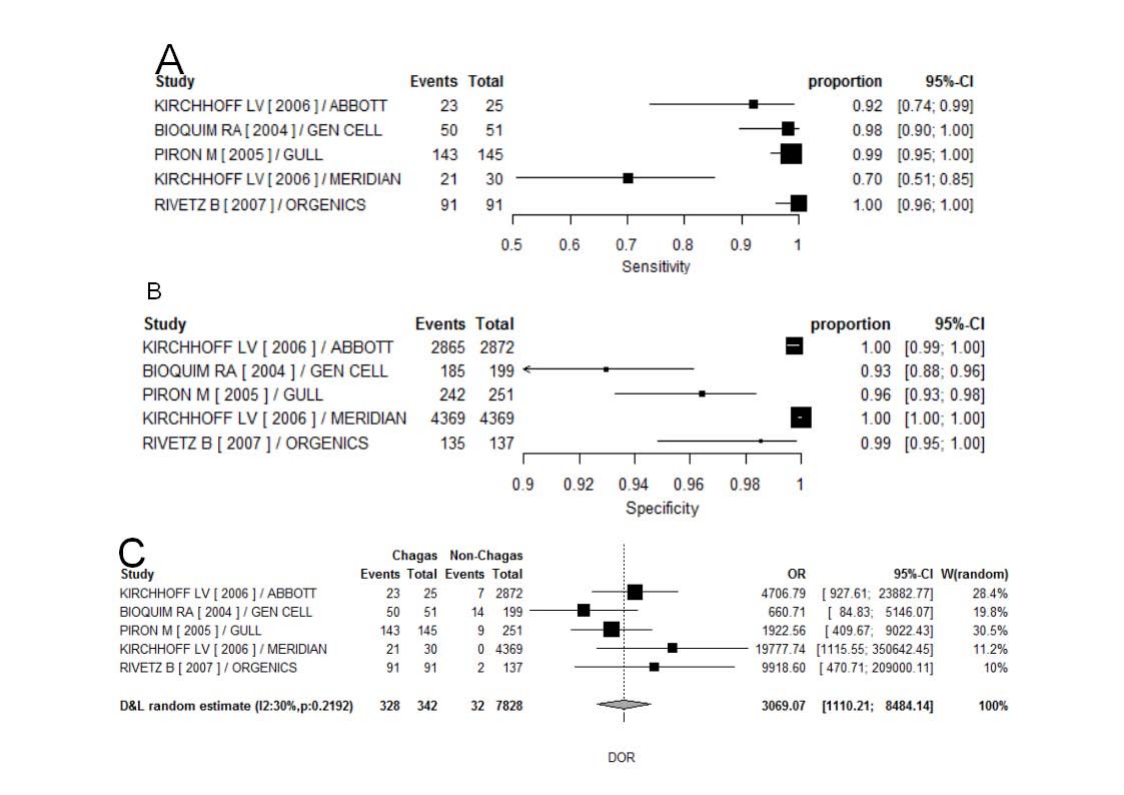
**Forest plots of sensitivity (A), specificity (B) and DOR (C) for phase 3 studies investigating commercial ELISA tests**. Events - identified as with Chagas disease by the test; OR - (diagnostic) odds ratio; proportion - sensitivity (or specificity) individual point estimate; Total - number of subject within each sample (with or without Chagas disease); W - weights.

Five commercial ELISA tests were classified as phase 3 and although there was high heterogeneity in sensitivity and specificity, it was moderate in DOR. (Additional file 5) Figure 3 shows forest plots of this group and the summary sensitivity was 94.3% and specificity was 99.9%. (Additional file 7)

**Figure 4:**
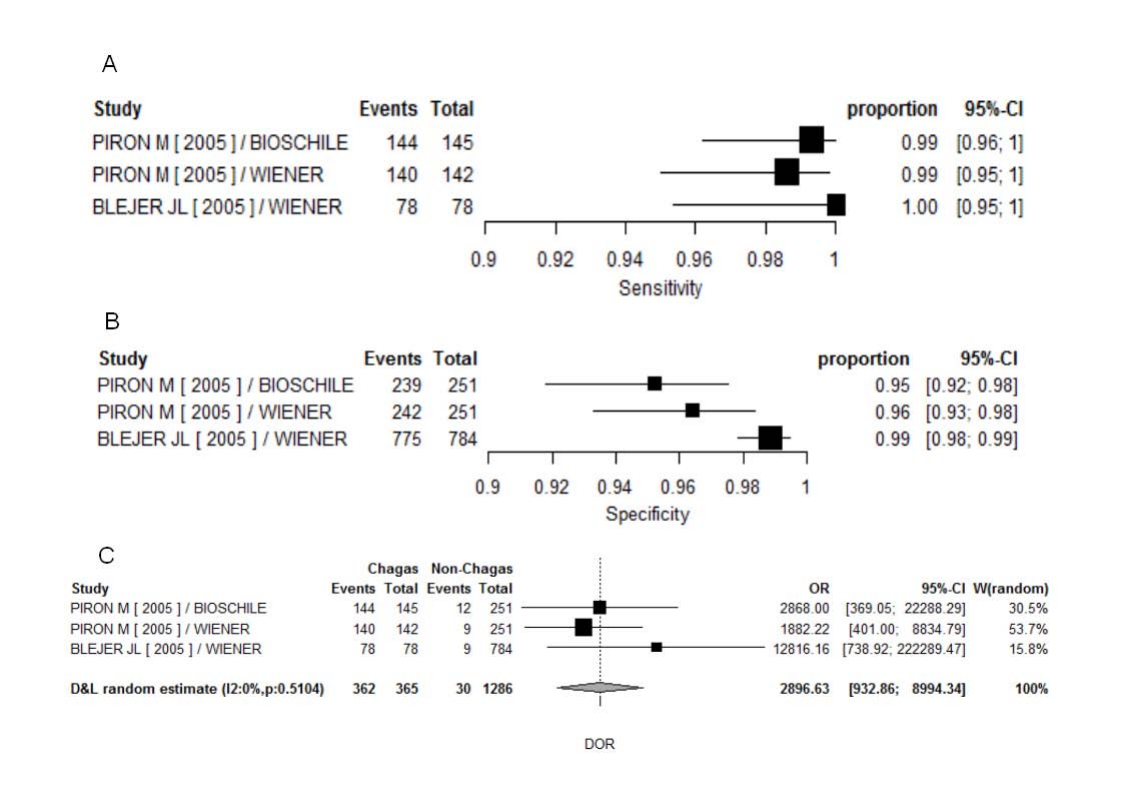
**Forest plots of sensitivity (A), specificity (B) and DOR (C) for phase 3 studies investigating commercial ELISA with recombinant antigens**. Events - identified as with Chagas disease by the test; OR - (diagnostic) odds ratio; Proportion - sensitivity (or specificity) individual point estimate; Total - number of subject within each sample (with or without Chagas disease); W - weights.

Similar to ELISA group, heterogeneity was very high in ELISA-rec. (Additional file 6). Within this group, only three commercial tests were classified as phase 3. In commercially available ELISA-rec tests investigated in phase 3 studies, heterogeneity was high in specificity, and low evidence was found in sensitivity and DOR. Threshold effect was not estimated due to difficulties of convergence of HSROC model, thus it was not considered appropriate. Summary estimates by bivariate random model are 99.2% and 97.5% for sensitivity and specificity respectively (Additional file 7). Forest plots for this subgroup are displayed in figure 4.

Analysis of a subgroup of commercial ELISA (regular and recombinant) investigated in phase 3 studies that used only blood donors in the sample demonstrated that only six studies fit these criteria. Heterogeneity was very high in all three measures and a threshold effect was not detected.

PCR has a below desired sensitivity (probably between 50% and 90%) and very high specificity (probably very close to 100%). Heterogeneity was very high and no variable explored was able to explain it. In the three validity estimates considered, I^2 ^is always over 70.0%. (Additional file 6) This means that from all available data, there was not a particular PCR feature that could explain the observed differences in PCR performance, thus pooling summary estimates by any PCR characteristics (such as DNA target region) was not considered appropriate. However, a threshold effect was detected in subgroups composed by two variables: test with samples stored with guanidine (yes or no) and boiled before processed (yes or no). In the three groups formed by this combination, the heterogeneity still remained very high in some pooled estimates. (Additional file 6) Sensitivity and specificity summaries are displayed in Additional file 7.

**Figure 5:**
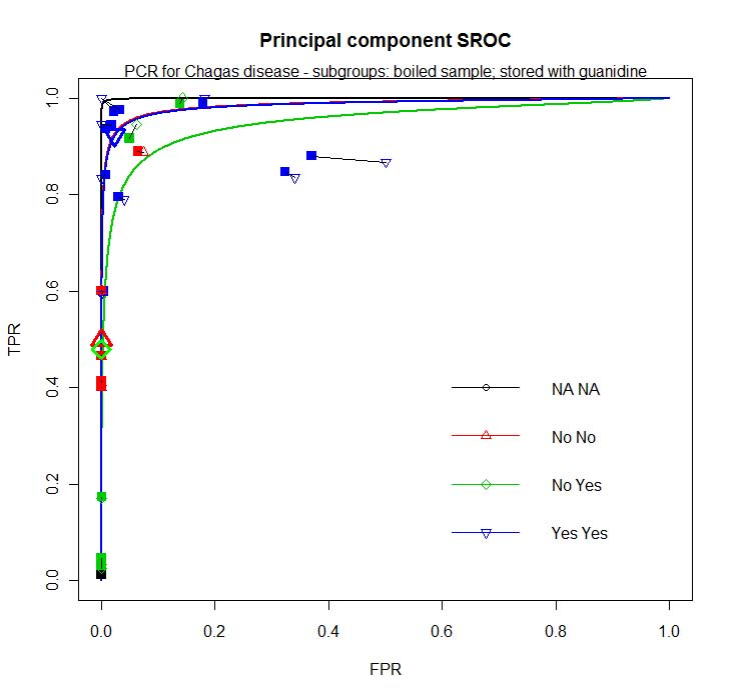
**PCR for Chagas disease HSROC - subgroups of blood samples boiled before processed and stored with guanidine**. Big blue upside down triangle - summary estimate of the group stored with guanidine and boiled; Big green diamond - summary estimate of the group stored with guanidine and not boiled; Big red triangle - summary estimate of the group not stored with guanidine and not boiled; Blue small squares and blue small upside down triangles - shrunken estimates from bivariate model and crude estimates (respectively) from group stored with guanidine and not boiled; blue solid line - SROC curve from group stored with guanidine and boiled; dot - crude estimates; small green squares and small green diamonds - shrunken estimates from bivariate model and crude estimates (respectively) from group stored with guanidine and not boiled; solid green line - SROC curve from group stored with guanidine and not boiled; small red squares and small red triangles - shrunken estimates from bivariate model and crude estimates (respectively) from group not stored with guanidine and not boiled; red solid line - SROC curve from group not stored with guanidine and not boiled; NA - not assigned or missing.

At first it seems that the group 'stored with guanidine and boiled' has a better performance, with sensitivity of 92.2% and specificity of 97.7%, while the other two groups have sensitivity close to 50% and specificity close to 100%. However, the summary curve of the former group is almost identical to the group 'not stored with guanidine and not boiled' while accuracy is lower for the group 'stored with guanidine and not boiled'. (figure 5) Also, it seems that all three curves have smaller areas when visually compared to the ELISA curve. (figure 2)

In the Chagas diagnostic literature there is a sense that reliability of serological tests is less than the desired level, however this review was able to identify only 7 investigations showing some result regarding reliability and in all of them the estimates are in acceptable levels (Additional file 1 and Additional file 2), and surprisingly no reliability was formally tested in any PCR investigation.

## Discussion

In the beginning of this review there was an interest in phase 3 investigations with commercially available tests, which are believed to have results more easily interpreted and are more readily available for use in clinical practice. However, the attempt to include technical reports from commercially available tests was not very successful, because only one non-published report was accessed and it seems that some commercial tests reports do not exist or are not accessible.

Recent Brazilian regulations states that medical products or devices do not need technical reports if they are used in vitro - for example, used on any patient's material such as blood, sputum, urine etc. - although they may be closely related to medical decision making. We wonder why technical reports are not available from regulatory register. Despite the reasons, their ability to correctly identify those with and without Chagas disease is unknown for those who use those tests in clinical practice.

Because of some perceptions during the review, such as: few phase three investigations; few commercial tests investigations sponsored by manufacturers; reports published mainly in immunology and parasitology journals; absence of products monographs or technical reports; as well as the amount of missing information is considerably higher in the commercial tests reports; it seems that in this field there is a considerable gap between the academic production, manufacturer interests and clinical practice.

Methodological quality of original reports was assessed through QUADAS tool. A careful reader would soon understand that QUADAS was developed to evaluate investigations in a more clinical scenario, for example, phase 3 investigations. Several issues such as gold standard description, inclusion and exclusion criteria and blinding assessment are perhaps less important in phase 2 than in phase 3, therefore the use of QUADAS could give a more strong impression of poor quality of the included investigations in this review, since the majority of original investigations were classified as phase 2.

The overall impression regarding methodological quality of included reports in this review is that poor quality of reporting is mixed with poor quality of investigations in most cases. This impression does not come solely from the level of "uncertainty" in QUADAS evaluation, but also from the amount of missing information about sample description and test description. Even in those investigations classified as phase 3, very few seem to follow current recommended standards for report formats [131].

Serological tests were seldom formally evaluated in phase three investigations with samples exclusively from blood donors. Although screening occurs in a different setting from diagnosis in clinical practice, the same tests are used and therefore the same problems are also found, such as high heterogeneity, and absence of a threshold effect. Particularly, in blood banks, the information about cut-off would be important because its variation may be a simple mean by which test accuracy could be improved. However, none of the phase three studies using exclusively blood donors described which test cut-off was used.

ELISA test results are generated as optical densities, which are presented as a continuous scale. However, none specified the area under the ROC curve (which is considered an accuracy measure independent from the cut-off used), and very few investigations used strategies to estimate a good cut-off considering both the distribution of results of those with and without the target disease, such as maximizing Youden's index. The majority of specified cut-off, when specified, was estimated by strategies such as 'mean of negative controls plus 2 standard deviations', which may maximize specificity but completely ignores sensitivity.

It is known that, similar to other infectious agents, there are differences of circulating strains in different geographic regions in Latin America [132]. Authors from Colombia,[95,133] Peru,[134] and Mexico [87] showed that commercial tests may have worst performances when compared to in-house tests with antigens made from local *T. cruzi *strains. There were some multicenter studies included in this review but the information about the antigen geographic source or stratified analysis by center was sparse, turning impossible to explore this issue as source of heterogeneity.

In Chagas diagnostic literature, researchers have repeatedly said that there is a lack of reliability or agreement of serological tests based on imperfect kappa estimated between different tests or laboratories [22-25,67,89,95,135]. Therefore, there is a difference of conceptions when comparing reliability definitions in general statistics[136] or diagnostic test methodology literature,[131] where instrument variability concerns the amount of variation that arises during the operation of devices or systems, such as automated laboratory measurements. Other terms for this form of variation include imprecision, reproducibility, analytic methodological variation, or analytical noise (error) and sometimes calibration [137]. Because this review did not find high intra-test variability of ELISA test, there is no evidence to support the lack of reliability statement.

It appears that there is no widely accepted PCR test protocol, since none of the tests found in literature used identical protocols. This may indicate that the PCR for *T. cruzi *is difficult to standardize and perhaps all protocols used are prone to some unacceptable procedure errors for clinical diagnosis. Most PCR protocols still use phenol-chloroform for DNA extraction instead of available commercial kits. (Additional file 3) Phenol-chloroform has biohazards issues and is no longer recommended for use in clinical laboratory routine or in hospital settings.

Also, primers aiming K-DNA were the most frequently used. Although these primers aim at conserved regions within K-DNA, the amplified region is considered hypervariable[138] leading to variations in the amplicon sizes. A possible problem with the K-DNA, which was seldom investigated, is that the primer annealing sequences are conserved within Kinestoplastida order [138]. However, sequence alignment of "121" and "122" analysis by comparing the "121" and "122" primers sequence with *T. rangeli *homologous region (GeneBank: http://www.ncbi.nlm.nih.gov - access number: L28038.1) showed that these primers cannot efficiently amplify the target from this specie due to mismatches in the 3' end of the primer, therefore the possibility of *T. rangeli *miss-amplification is remote.

The different primer sequences used in the molecular tests could be responsible for the observed test heterogeneity. There was seven different set of primers used in the twenty one included studies. Most studies applied primer sequences targeted to K-DNA and ns-DNA. The intention is to increase analytical sensitivity, as these are repetitive regions in *T. cruzi *DNA. Also, primers for K-DNA are slight modifications from one another within the same target region, which suggests that these primers may have limitations for *T. cruzi *DNA amplification.

It was not possible to explore the observed variation of primers as source of heterogeneity due to the number of groups that used different sets of primers, and some primers were used only once. This review did not find any evidence of superior analytical sensitivity of a particular primer while most of the included studies did not report this information.

Some authors stated that there is no clear advantage and there are some disadvantages in using guanidine over EDTA before extraction [139,140]. In hospital settings where samples could be processed, and DNA could be extracted in the very same day, perhaps this would no longer be necessary. Boiling the blood sample before DNA extraction was also commonly described. It has been shown that boiling during 15 minutes disrupts the K-DNA minicircle nets, thus facilitating the homogeneous distribution of minicircle molecules in all the volume of Guanidine-EDTA treated sample and improving primer annealing to the templates, thus allowing processing of small volumes with high sensitivity. However, his review did not find evidence that this procedure increases PCR performance.

PCR success depends on the amount of circulating parasites in patients' blood stream. *T. cruzi *circulates in very small amounts at the chronic phase and dynamics about its circulation is not predictable [141,142]. It is possible that, even if a patient is infected, the collected sample does not have an adequate amount of the parasite DNA leading the test to a negative or undetectable result. A possible solution to this limitation is the collection of several serial blood samples at different times[126] or increasing the blood volume per test may overcome this problem.

Although this review has not intended to evaluate any test to assess parasitological cure of Chagas disease, in recent literature addressing this issue there is much discussion about PCR techniques for detecting trypanocidal therapy outcome, primarily the real-time PCR [143,144]. This reflects the fact that serological markers and clinical disease progression may take decades to be observed. However, this review found no evidence that PCR tests are adequate to correctly identify (mainly) the presence of *T. cruzi *DNA. Perhaps PCR tests could be a suitable tool to detect therapy outcome, in particular therapy failure, but this remains to be evaluated in prospective studies.

In 2007, TDR (a Special Program for Research and Training in Tropical Diseases) launched an international multicenter study to standardize PCR procedures with a panel of samples. The results of such study were analyzed in a workshop with experts in which consensus recommendations to run PCR for *T. cruzi *were formulated. This can be assessed in http://apps.who.int/tdr/svc/publications/tdrnews/issue-82/meeting-chagas. Its clinical usefulness, however still remains to be evaluated.

## Review limitations

The main limitations of this review are: (1) inability to find unpublished technical reports from tests commercially available, therefore this review may not represent all ELISA tests or might have biased results; (2) summary estimates were pooled from selected subgroups where heterogeneity was partially explained, therefore interpretation of results are less straight forward then a result where heterogeneity is absent.

The quality of a systematic review is also influenced by the original investigations' quality. It is likely that other items not explored by this review could explain the observed heterogeneity. Examples of these items are: data collection period; proportion of children in sample; sex distribution in sample; sample median age; age range; if volunteers were from rural or urban area; geographical origin of volunteers; Chagas disease clinical presentation such as cardiac or intestinal involvement; ELISA generation; methods of preparations of antigens, buffers or brand of plates; type of antibodies used; how cut-off was estimated; indeterminate range; *T. cruzi *life cycle form (epimastigote, trypomastigote etc) used as source of antigen; geographical areas of the strains used as source of antigens; inhibition control for PCR, contamination control for PCR, time gap between sample collection and DNA extraction, maintenance condition of the sample; polymerase trademarks; type of hybridization technique (colorimetric or radioactive); primer variation; inhibition

Many of these characteristics were not explored as heterogeneity source because of the amount of original investigations that did not report them, but other made sensitive analysis difficult because many subgroups had a single study.

## Conclusions

ELISA and ELISA-rec performances are good. Their reliability is within acceptable ranges although not often studied. These findings lead to the conclusion that recommendations to use two simultaneous serological tests is based on mistrust in recommending a single test that will fail very occasionally, or on a misunderstanding of the reliability concept of diagnostic tests. Both ELISA and ELISA-rec could be used as a single test for chronic Chagas disease diagnosis, but caution is necessary while some commercial tests technical reports were not assessed by this review, thus they were not included and their performance are not known.

This review results about PCR test are less conclusive then ELISA, thus more difficult to interpret. Besides strong evidence of heterogeneity, only one study was classified as phase 3. Currently PCR performance is below desired and its reliability has not been characterized. Visual comparison of the area under the summary ROC curves for ELISA and PCR indicates that ELISA has better performance than PCR. At this point, PCR test cannot be considered a tool for diagnosis of chronic Chagas disease in clinical practice.

## Final considerations

There are many investigations about chronic Chagas disease diagnosis since 1980, however little knowledge reached clinical practice with current recommended standards up to 2009. Phase 3 investigations and commercial tests detailed reports are necessary, and they should follow standard report format,[131] always making explicit: test reliability (intra-test agreement); if reference standard and index test were blinded to each other; volunteer selection strategy, mainly if it was based on clinical suspicion of disease or not; clear description of clinical characteristics of the volunteers; the cut-off used and indeterminate range of the test and how they were estimated; also, always include key issues of test protocol, such as strains used in antigens development, or which strains would the recombinant antigens could represent, and antigen purification. Regulatory agencies would make a great step forward if diagnostic tests' technical reports would be always necessary for product registry and always available to the public.

Besides the quality of reports and design of diagnostic research, there are points that could be addresses to improve tests in the future. Serological tests could have a set of recombinant or crude antigens combination which could equally detect different *T. cruzi *strains from distant geographic areas, in order to have similar performance with patients from different locations. The development and improvement of strip tests, or portable tests, may be very useful, while the majority of patients may be at distant rural areas where health care access is difficult. Also, Chagas disease pathophysiolgy further understanding may help to improve serological tests while these tests aim to detect patient's antibodies, thus there are patients' characteristics that may influence serological tests performances.

Concerning PCR, much more is yet to be done. Incorporation of modern techniques already available for PCR to other diseases diagnosis (for example: HBV, HIV and tuberculosis), such as commercial DNA extraction kits, real-time and other techniques that could make the test more automated should be encouraged. In addition, research on updated DNA sequence for PCR primers design and use of multiple primer sets including multiplex PCR test and primers aiming to parasite's DNA inserted into host genome should be encouraged. Also, it is expected that performing a serial tests in a patient and defining the diagnosis after assembling all the results would increase test sensitivity.

## Competing interests

The authors declare that they have no competing interests.

## Authors' contributions

PEAAB carried out the design, coordination, project/protocol development, review, data analysis, draft development, LDeC was a reviewer and draft reviewer, AMHM was a reviewer and draft reviewer, LHCS was a reviewer and draft reviewer, JUB was involved in project development and draft review. All authors read and approved the final manuscript.

## Pre-publication history

The pre-publication history for this paper can be accessed here:

http://www.biomedcentral.com/1471-2334/10/337/prepub

## Supplementary Material

Additional file 1 **ELISA for chronic Chagas disease descriptive summary - test characteristics; population/sample characteristics and test validity measures**. 2/3-: two negative tests out of three; 2/3+: two positive tests out of three; CF or CFR: complement fixation reaction; CML: complement mediated lyses; CO Method: method to estimate the cut-off; DA: direct agglutination; ELISA: enzyme linked immunosorbent assay; Exclusively BB: investigation sample composed exclusive by blood donors; Gray zone: test scale inconclusive range; IHA and HA: (indirect) hemmagglutination; IIF: indirect immunefluorescence; IPR: immuneperoxidase reaction; -MEAN or +MEAN: mean values from those without or with Chagas disease respectively; NA: not assigned or missing; OD: optical densities; PHA: passive hemagglutination; Reliability: intra-test agreement; RIPA: radio-immune-precipitation assay; ROC: receiver operator characteristic; RPHA: reverse passive hemagglutination; RS1 and RS0: reference standard for subjects classified with and without Chagas disease respectively; SD: standard deviation; SE: standard error; WB: western blot; year: year of publication Click here for file

Additional file 2 **ELISA with recombinant antigens for chronic Chagas disease descriptive summary - test characteristics; population/sample characteristics and test validity measures**. 2/3-: two negative tests out of three; 2/3+: two positive tests out of three; CF or CFR: complement fixation reaction; CO Method: method to estimate the cut-off; Exclusively BB: investigation sample composed exclusive by blood donors; Gray zone: range of test scale where results are considered inconclusive; IHA and HA: (indirect) hemmagglutination; IIF: indirect immunefluorescence; -MEAN or +MEAN - mean values from those without or with Chagas disease respectively; NA: not assigned or missing; OD: optical densities; PA: passive agglutination; Reliability: intra-test agreement; RIPA: radio-immune-precipitation assay; RS1 and RS0: reference standard for subjects classified with and without Chagas disease respectively; SD: standard deviation; SE: standard error; year: year of publication. Click here for file

Additional file 3 **PCR for Chagas disease descriptive summary - test characteristics; population/sample characteristics and test validity measures**. 2/2-: two negative tests out of two; 2/2+: two positive tests out of two; ELISA - enzyme linked immunosorbent assay; Exclusively BB - investigation sample composed exclusive by blood donors; HA - hemagglutination; IIF - indirect immunefluorescence; K-DNA - kinetoplast deoxyribonucleic acid; NA - not assigned or missing; ns-DNA: nuclear satellite DNA; PCR - polymerase chain reaction; Reliability: intra-test agreement; RS1 and RS0 - reference standard for subjects classified with and without Chagas disease respectively; year - year of publication. Click here for file

Additional file 4 **Quality assessment with QUADAS tool of all tests evaluated**. Reader must refer to QUADAS full questionnaire for a comprehensive interpretation of this table. Click here for file

Additional file 5 **Primers used in PCR tests in each of the original investigations reviewed**. *Intermediate products. Click here for file

Additional file 6 **Heterogeneity (I^2^) estimates and its 95% confidence limits for ELISA, ELISA-rec, PCR and selected subgroups**. DOR - diagnostic odds ratio; ELISA - enzyme linked immunosorbent assay; ELISA-rec - ELISA with recombinant antigens; inf cl - inferior confidence limit; N - number of tests included in subgroups; PCR - polymerase chain reaction; Se - sensitivity; Sp Specificity; sup cl - superior confidence limit. Click here for file

Additional file 7 **Summary Sensitivity and Specificity estimated by bivariate random model in selected subgroups**. ( ): number of tests in each group; FPR: False positive rate or 1-Specificity; MCMC: Monte Carlo Markov Chain; ML: Maximum likelihood; Reff: Random effect; SD: Standard deviation; TPR: True positive rate or Sensitivity; upper and lower: 95% confidence limits. Click here for file

Additional file 8 **List of excluded papers and comments about reasons of exclusions**. Click here for file

Additional file 9 **Chart with strategies used on remote databases search**. Click here for file
