# Supplementary material for: ELISA versus PCR for diagnosis of chronic Chagas disease: systematic review and meta-analysis
Source: BMC Infect Dis. 2010 Nov 25;10:337. doi: 10.1186/1471-2334-10-337 (PMC3004908; doi:10.1186/1471-2334-10-337)
Supplement: Additional file 8 — List of excluded papers and comments about reasons of exclusions. [file 1471-2334-10-337-S8.DOCX]

**Excluded articles and reasons.**

(1) Sort of editorial about blood banks screening and the introduction of screening routine for Chagas disease. ID = 3

(2) Very preliminary phase of investigation. Only descriptive procedures. No data for extraction. ID = 14

(3) There is undefined terms such as co-positivity. There is more than one reference standard and no clear comparison with any of them. ID = 19

(4) Not possible to separate data from acute and chronic Chagas disease. ID=28

(5) There is no explicit data for extraction although a graphical result is presented. Very preliminary phase to identify possible antigens. ID = 39

(6) There is no data for extraction. ID = 41

(7) Same author published same data in another report. ID = 84

(8) Same author published same data in another report. ID = 85

(9) Same author published same data in another report. ID = 87

(10) Sample does not have subjects with and without Chagas disease. ID = 103

(11) Does not study ELISA. ID = 118

(12) Does not study ELISA. ID = 133

(13) Patients with Chagas disease are a control group for a Leishmaniasis serological study. ID = 158

(14) It is not possible to extract data for those without Chagas disease. It seems that the information in tables does not match with information in the text. ID = 162

(15) Does not study ELISA. ID = 168

(16)Does not study ELISA. ID = 177

(17) It is not possible to separate the chronic patients data from those with acute disease. ID = 185

(18) Proficiency study. ID = 189

(19) Sample does not study patients without Chagas disease. ID = 191

(20) Does not study ELISA. ID = 199

(21) Data from patients without Chagas disease are not clear. ID = 219

(22) It seems like a proficiency study. Explores different recombinant antigens but does not have clear data for accuracy. ID = 246

(23) No data for extraction. ID = 248

(24) 38% of the sample was not tested by the reference standard. ID = 255

(25) Does not investigate ELISA. ID = 274

(26) ELISA is used as the reference standard. ID = 278

(27) Very preliminary phase 1 diagnostic study design, no data for extraction. ID = 286

(28) No data for extraction. ID = 295

(29) No data for extraction. ID = 309

(30) Results from treated and non treated patients are mixed. ID = 312

(31) Same author published same data in another report. ID = 350

(32) Very preliminary phase 1, it is not clear if samples are from human. ID = 399

(33) Not possible to separate data form children (acute) and adults (chronic). ID = 400

(34) Review. ID = 412

(35) Proficiency study. ID = 415

(36) Does not define a reference standard. Data for extraction is not clear. ID = 423

(37) Data for extraction is not clear. Only 4 patients with Chagas disease. ID = 425

(38) HCV investigation where some patients with Chagas disease are used as controls. ID = 454

(39)No clear definition of a reference standard. Results and discussion run over agreement between tests. ID = 472

(40)Very preliminary phase one study. No clear data for extraction. ID = 504

(41)Data from treated and non treated patients are mixed. ID = 509

(42) Data from patients without Chagas disease are not clear. ID = 511

(43) The sera samples from those without disease were used only to estimate a cut-off. Data from tables don’t seem to match data from text. ID = 533

(44) Expert opinion. ID = 534

(45) No clear data for extraction. ID = 581

(46) 91% of the sample was not submitted to a reference standard. ID = 589

(47) There is no data from patients without Chagas disease. ID = 638

(48) No clear data for extraction. ID = 639

(49) Does not estimate accuracy, only agreement and reliability although not clearly defined. ID = 672

(50) Review. ID = 679

(51) Proficiency study. ID = 680

(52) Proficiency study. ID = 681

(53) Does not study ELISA. ID = 682

(54) Does not study ELISA. ID = 709

(55) Does not study ELISA. ID = 711

(56)Does not study humans subjects, neither has samples representing those with and without Chagas disease. It is not a quantitative investigation. ID = 713

(57)Does not study humans subjects, neither has samples representing those with and without Chagas disease. It is not a quantitative investigation. Not original work. ID = 714.

(58) Not ELISA. ID = 718.

(59)Aim to identify possible molecules with mixed infection. Not quantitative. ID = 719.

(60)Early phase 1 study. No binary data for extraction. ID = 720.

(61)Proficiency study. ID = 721.

(62)Not ELISA. ID = 722.

(63)Not ELISA. ID = 723.

(64)Not with human beings. ID = 725.

(65)No data for extraction. ID = 726.

(66)Patients with Chagas used as controls for Leishmania tests. ID = 727.

(67)Not ELISA. ID = 728.

(68)Does not study ELISA accuracy, no data for extraction. ID = 731.

(69)Not ELISA. ID = 733.

(70) Not ELISA. ID = 734.

(71)Proficiency study. ID = 736

(72) Not ELISA. ID = 737.

(73) Not ELISA. ID = 738.

(74) Not ELISA. ID = 739.

(75) Not ELISA, and there is no subjects without Chagas disease. ID = 740.

(76) Not ELISA. ID = 742.

(77) Not ELISA. ID = 743.

(78) Not quantitative research, there is no human subjects. ID = 744.

(79)Not ELISA. ID = 745

(80) There are no subjects with and without Chagas disease. ID = 750.

(81)41% of the sample was inconclusive by the reference standard. ID = 752.

(82)No separated data for ELISA. ID = 753.

(83) Review. ID = 755.

(84)Not ELISA. ID = 756.

(85)Not ELISA. ID = 757.

(86) Not ELISA. ID = 759.

(87) It is not a quantitative research and there are no subjects without Chagas disease. ID = 760

(88)No patients without Chagas disease. Only analytical measures. ID = 762.

1. Blood donor screening for chagas disease--United States, 2006-2007. MMWR Morb Mortal Wkly Rep. 2007 Feb 23;56(7):141-3.

2. Almeida IC, Rodrigues EG, Travassos LR. Chemiluminescent immunoassays: Discrimination between the reactivities of natural and human patient antibodies with antigens from eukaryotic pathogens, Trypanosoma cruzi and Paracoccidioides brasiliensis. J Clin Lab Anal. 1994;6(8):424-31.

3. Alvarez F, Montagnani S, Devesa M, Contreras I. Elisa para chagas en formato de palillo. Acta cient Soc Venez Bioanalistas Esp. 1993;2(1):31.

4. Anez N, Crisante G, Rojas A, Carrasco H, Parada H, Yepez Y, et al. Detection and significance of inapparent infection in Chagas disease in western Venezuela. Am J Trop Med Hyg. 2001 Sep;65(3):227-32.

5. Araujo FG. Analysis of Trypanosoma cruzi antigens bound by specific antibodies and by antibodies to related trypanosomatids. Infect Immun. 1986 Jul;53(1):179-85.

6. Araujo FG, Guptill D. Use of antigen preparations of the amastigote stage of Trypanosoma cruzi in the serology of Chagas' disease. Am J Trop Med Hyg. 1984 May;33(3):362-71.

7. Blejer JL, Saguier MC, Salamone HJ. Antibodies to Trypanosoma cruzi among blood donors in Buenos Aires, Argentina. Int J Infect Dis. 2001;5(2):89-93.

8. Blejer JL, Saguier MC, Salamone HJ. Anticuerpos anti-Trypanosoma cruzi en donantes de sangre. Rev argent transfus. 2004;30(3/4):203.

9. Blejer JL, Saguier MC, Dinapoli RA, Salamone HJ. Prevalence of anti-Trypanosoma cruzi antibodies in blood donors. Medicina (B Aires). 1999;59(2):129.

10. Breniere SF, Poch O, Selaes H. Specific humoral depression in chronic patients infected by Trypanosoma cruzi. Revista do Instituto de Medicina Tropical de Sao Paulo. 1984;26(5):254.

11. Buchovsky AS, Campetella O, Russomando G, Franco L, Oddone R. Candia N, Luquetti A, et al. trans-Sialidase inhibition assay, a highly sensitive and specific diagnostic test for Chaga's disease. Clin Diagn Lab Immunol. 2001;8(1):187.

12. Carlier Y, Breniere FS, Lemesre LJ, Carrasco R, Desjeux P, Afchain D. The interest of immunoprecipitation tests in the immunological diagnosis of Chagas' disease. Ann Soc Belg Med Trop. 1985;65 Suppl 1:85-94.

13. Chiaramonte MG, Zwirner NW, Caropresi SL, Heredia V, Taranto NJ, Malchiodi EL. Human leishmaniasis infection in the province of Salta. Evidence of mixed infection with Trypanosoma cruzi and Leishmania spp. Med-Buenos Aires. [Article]. 1996;56(3):259.

14. Coelho JS, Soares IDS, de Lemos EA, Jimenez MC, Kudo ME, Moraes SD, et al. A multianalyte Dot-ELISA for simultaneous detection of malaria, Chagas disease, and syphilis-specific IgG antibodies. Diagn Microbiol Infect Dis. 2007 Jun;58(2):223-30.

15. Cordeiro FD, Martins-Filho OA, Da Costa Rocha MO, Adad SJ, Corre?a-Oliveira R, Romanha AJ. Anti-Trypanosoma cruzi immunoglobulin G1 can be a useful tool for diagnosis and prognosis of human Chagas' disease. Clin Diagn Lab Immunol. 2001;8(1):112.

16. Cuna WR, Rodriguez C, Torrico F, Afchain D, Loyens M, Desjeux P. Evaluation of a competitive antibody enzyme immunoassay for specific diagnosis of Chagas' disease. J Parasitol. 1989 Jun;75(3):357-9.

17. Lopez-Chejade P, Fisa R, Gallego M, Iniesta L, Riera C, Portus M. Chagas disease diagnosis in population from endemic countries in Barcelona. Evaluation of the diagnostic tool. [Spanish]. Enfermedades Emergentes. [Conference Paper]. 2005;SUPPL. 1(7):32-4.

18. Lorca M, Child R, Garcia A, Silva M, Martinez L, Jerez G, et al. Assessment Of Commercial Kits Used For Chagas-Disease Detection In Blood-Banks.2. Routine Application. Rev Medica Chile. [Article]. 1994 Aug;122(8):925.

19. Lorca M, Gonzalez A, Reyes V, Veloso C, Vergara U, Frasch C. Diagnosis Of Chronic Chagas-Disease Using Recombinant Trypanosoma-Cruzi Antigens. Rev Medica Chile. [Article]. 1993 Apr;121(4):363.

20. Luquetti AO, Ponce C, Ponce E, Esfandiari J, Schijman A, Revollo S, et al. Chagas' disease diagnosis: a multicentric evaluation of Chagas Stat-Pak, a rapid immunochromatographic assay with recombinant proteins of Trypanosoma cruzi. Diagn Microbiol Infect Dis. 2003 Aug;46(4):265-71.

21. Marcelain K, Colombo A, Molina MC, Ferreira L, Lorca M, Aguillo?n JC, et al. Development of an immunoenzymatic assay for the detection of human antibodies against Trypanosoma cruzi calreticulin, an immunodominant antigen. Acta Tropica. 2000;75(3):291.

22. Monteon VM, Guzman-Bracho C, Floriani-Verdugo J, Ramos-Echevarria A, Velasco-Castrejon O, Reyes PA. [Serological diagnosis of Chagas disease: self-sufficiency and interlaboratory concordance]. Salud Publica Mex. 1995 May-Jun;37(3):232-5.

23. Monteón VMP, Ramos AE, Reyes PA. Reactividad de sueros de pacientes chagásicos crónicos con extractos de aislamientos mexicanos de Trypanosoma cruzi. Rev biol trop. 1993;41(3B):861.

24. Moret AL, Saporiti MP, Ascione A, Bustos D. Serodiagnosis of Chagas's disease. Utility of an enzyme immunoassay with recombinant antigen on samples with inconclusive results. Acta Bioquim Clin Latinoam. [Article]. 2003 Jun;37(2):153.

25. Neto VA, De Marchi CR, Ferreira CS, Ferreira AW. Observations on the use of TESA blot for the serological diagnosis of Chagas' disease. [Portuguese]. Rev Soc Bras Med Trop. 2005;6(38):534-5.

26. Nilsson LA, Voller A. A comparison of thin layer immunoassay (TIA) and enzyme-linked immunosorbent assay (ELISA) for the detection of antibodies to Trypanosoma cruzi. Trans R Soc Trop Med Hyg. 1982;76(1):95-7.

27. O'Daly JA, Carrasco H, Fernandez V, Rodriguez MB. Comparison of chagasic and non-chagasic myocardiopathies by ELISA and immunoblotting with antigens of Trypanosoma cruzi and Trypanosoma rangeli. Acta Trop. 1994 Apr;56(4):265-87.

28. Orozco LC, Camargo D, López MC, Duque S, Gualdrón LE, Cáceres E, et al. Inmunodiagnóstico de la infección en humanos por Trypanosoma cruzi mediante Elisa utilizando sangre recolectada en papel de filtro. Biomédica (Bogotá). 1999;19(2):164.

29. Paranhos-Bacalla GS, Santos MR, Cotrim PC, Rassi A, Jolivet M, Camargo ME, et al. Detection of antibodies in sera from Chagas' disease patients using a Trypanosoma cruzi immunodominant recombinant antigen. Parasite Immunol. 1994 Mar;16(3):165-9.

30. Passos VM, Volpini AC, Braga EM, Lacerda PA, Ouaissi A, Lima-Martins MV, et al. Differential serodiagnosis of human infections caused by Trypanosoma cruzi and Leishmania spp. using ELISA with a recombinant antigen (rTc24). Mem Inst Oswaldo Cruz. 1997 Nov-Dec;92(6):791-3.

31. Umezawa ES, Bastos SF, Camargo ME, Yamauchi LM, Santos MR, Gonzalez A, et al. Evaluation of recombinant antigens for serodiagnosis of Chagas' disease in south and central America. J Clin Microbiol. [Article]. 1999 May;37(5):1554.

32. Zanoni TB, Carlos IZ, Tognolli JO, Yamanaka H, Ferreira AAP. Optimization of ELISA using Tc85-11 protein and factorial design. Eclética Química. 2006;31(1):63.

33. Zarate-Blades CR, Blades N, Nascimento MS, da Silveira JF, Umezawa ES. Diagnostic performance of tests based on Trypanosoma cruzi excreted-secreted antigens in an endemic area for Chagas' disease in Bolivia. Diagn Microbiol Infect Dis. 2007 Feb;57(2):229-32.

34. da Silveira JF, Umezawa ES, Luquetti AO. Chagas disease: recombinant Trypanosoma cruzi antigens for serological diagnosis. Trends Parasitol. 2001 Jun;17(6):286-91.

35. D'Agostino L, Illa C, Mazziotta D. External quality evaluation for serological diagnosis of Chagas' disease. Evaluacio?n externa de calidad para el diagno?stico serolo?gico de la enfermedad de Chagas. 2002;36(4):663.

36. de Hubsch RM, Chiechie N, Comach G, Aldao RR, Gusmao RD. [The Dot immunoenzymatic assay on nitrocellulose (Dot-ELISA) in the diagnosis of Chagas disease. II. Seroepidemiologic study in 4 rural communities of Venezuela]. Mem Inst Oswaldo Cruz. 1989 Jul-Sep;84(3):401-8.

37. De Lima Rivero AR, Farias Tamoy MN, Tortolero Leal E, Navarro Aguilera MC, Contreras Alvarez VT. [Partial purification and use of Trypanosoma cruzi glycosidic fractions for Chagas disease diagnosis]. Acta Cient Venez. 2001;52(4):235-47.

38. dos Santos VA, Azevedo RS, Camargo ME, Alves VAF. Serodiagnosis of hepatitis C virus - Effect of new evaluation of cutoff values for enzyme-linked immunosorbent assay in Brazilian patients. Am J Clin Pathol. [Article]. 1999 Sep;112(3):418.

39. Ferreira AW, Belem ZR, Moura ME, Camargo ME. [Standardization of serological tests for Chagas disease: an immunoenzymatic test for blood donors triage]. Rev Inst Med Trop Sao Paulo. 1991 Mar-Apr;33(2):123-8.

40. Gea S, Rodriguez P, Vottero-Cima E. Characterization of Trypanosoma cruzi antigen recognized by sera from patients with chronic Chagas' disease. International Archives of Allergy and Applied Immunology. 1987;4(84):410-3.

41. Girones N, Rodriguez CI, Basso B, Bellon JM, Resino S, Munoz-Fernandez MA, et al. Antibodies to an epitope from the Cha human autoantigen are markers of Chagas' disease. Clin Diagn Lab Immunol. 2001;8(6):1039.

42. Godsel LM, Tibbetts RS, Olson CL, Chaudoir BM, Engman DM. Utility of recombinant flagellar calcium-binding protein for serodiagnosis of Trypanosoma cruzi infection. J Clin Microbiol. 1995 Aug;33(8):2082-5.

43. Guevara AG, Taibi A, BillautMulot O, Ouaissi A. Trypanosoma cruzi: A 6 x histidine-fused Tc24 protein useful for the serological diagnosis of Chagas' disease. Medical Science Research. [Article]. 1997 Jun;25(6):399.

44. Guhl F. Purified Trypanosoma cruzi specific glycoprotein for discriminative serological diagnosis of South American trypanosomiasis (Chagas' disease). Mem Inst Oswaldo Cruz. 1990 Oct-Dec;85(4):531-2.

45. Krieger MA, Almeida E, Oelemann W, Lafaille JJ, Pereira JB, Krieger H, et al. Use of recombinant antigens for the accurate immunodiagnosis of Chagas' disease. Am J Trop Med Hyg. 1992 Apr;46(4):427-34.

46. Larrouy G, Brochier B, Dos Santos LG, Queiroz SF, Magnaval JF. [ELISA methods for the detection of Trypanosoma cruzi carriers. Comparative study]. Bull Soc Pathol Exot Filiales. 1983 Nov;76(5):553-8.

47. Tachibana H, Kawabata M, Mimori T, Hashiguchi Y, Nagakura K, Kaneda Y. The validity of serodiagnosis using a monoclonal antibody against Trypanosoma cruzi-specific Mr 25,000 antigen for chagasic patients without cardiomyopathy. Ann Trop Med Parasitol. 1991 Apr;85(2):275-6.

48. Tachibana H, Nagakura K, Kaneda Y. Serodiagnosis of Chagas' disease using monoclonal antibody against Trypanosoma cruzi-specific Mr 25,000 antigen. Parasitol Res. 1988;74(5):409-14.

49. Ross A, Novoa-Montero D. Comparability and reliability of ELISA, immunofluorescence, and indirect hemagglutination assays for Trypanosoma cruzi and Trypanosoma rangeli. J Infect Dis. 1993 Dec;168(6):1581-4.

50. Saez-Alquezar A. Tamizaje y diagnóstico serológico de la infección por Trypanosoma cruzi. Rev argent transfus. 2003;29(3/4):117.

51. Saez-Alquezar A, Murta M, Pereira Marques W, Da Silva GR. The results of an external quality control program for serological screening for antibodies against Trypanosoma cruzi in blood donors in Brazil. [Spanish]. Revista Panamericana de Salud Publica/Pan American Journal of Public Health. 2003;2-3(13):129-37.

52. Saez-Alquezar A, Otani MM, Sabino EC, Ribeiro-dos-Santos G, Salles N, Chamone DF. Evaluation of the performance of Brazilian blood banks in testing for Chagas' disease. Vox Sanguinis. 1998;74(4):228.

53. Saez-Alquezar A, Sabino EC, Salles N, Chamone DF, Hulstaert F, Pottel H, et al. Serological confirmation of Chagas' disease by a recombinant and peptide antigen line immunoassay: INNO-LIA Chagas. J Clin Microbiol. 2000;38(2):851.

54. Moncayo A, Luquetti AO. Multicentre double blind study for evaluation of Trypanosoma cruzi defined antigens as diagnostic reagents. Mem Inst Oswaldo Cruz. 1990 Oct-Dec;85(4):489-95.

55. Zingales B, Gruber A, Ramalho CB, Umezawa ES, Colli W. Use of two recombinant proteins of Trypanosoma cruzi in the serological diagnosis of Chagas disease. Mem Inst Oswaldo Cruz. 1990 Oct-Dec;85(4):519-22.

56. Travassos LR, Almeida IC, Milani SR, Gorin PAJ. Antibody specificity in the conventional serology for Chagas disease. Ci Cult J Braz Assoc Adv Sci. 1993;45:69-73.

57. Frasch AC, Reyes MB. Diagnosis of Chagas disease using recombinant DNA technology. Parasitol Today. 1990 Apr;6(4):137-9.

58. Lemesre JL, Afchain D, Orozco O, Loyens M, Breniere FS, Desjeux P, et al. Specific and Sensitive Immunological Diagnosis of Chagas' Disease by Competitive Antibody Enzyme Immunoassay Using a Trypanosoma cruzi-Specific Monoclonal Antibody. Am J Trop Med Hyg. 1986;35(1):86-93.

59. Levy Yeyati P, Bonnefoy S, Mirkin G, Debrabant A, Lafon S, Panebra A, et al. The 70-kDa heat-shock protein is a major antigenic determinant in human Trypanosoma cruzi/Leishmania braziliensis braziliensis mixed infection. Immunol Lett. 1992 Jan;31(1):27-33.

60. Guimaraes MCS, Celeste BJ, de Castilho EA, Mineo JR, Diniz JMP. Immunoenzymatic Assay (Elisa) in Mucocutaneous Leishmaniasis, Kala-Azar, and Chagas' Disease: An Epimastigote Trypanosoma cruzi Antigen Able to Distinguish between Anti-Trypanosoma and Anti-Leishmania Antibodies. Am J Trop Med Hyg. 1981;30(5):942-7.

61. Luquetti AO. Use of Trypanosoma cruzi defined proteins for diagnosis--Multicentre trial. Serological and technical aspects. Mem Inst Oswaldo Cruz. 1990 Oct-Dec;85(4):497-505.

62. Lorca M, Gonzalez A, Veloso C, Reyes V, Vergara U. Immunodetection of antibodies in sera from symptomatic and asymptomatic Chilean Chagas' disease patients with Trypanosoma cruzi recombinant antigens. Am J Trop Med Hyg. 1992 Jan;46(1):44-9.

63. Andrews NW, Einstein M, Nussenzweig V. Presence of antibodies to the major surface glycoprotein of Trypanosoma cruzi amastigotes in sera from Chagasic patients. Am J Trop Med Hyg. 1989 Jan;40(1):46-9.

64. Gonzalez A, Prediger E, Huecas ME, Nogueira N, Lizardi PM. Minichromosomal Repetitive DNA in Trypanosoma cruzi: Its Use in a High-Sensitivity Parasite Detection Assay. Proceedings of the National Academy of Sciences. 1984;81(11):3356-60.

65. Teixeira AR, Pereira LM. Discrepancias entre resultados de tres reacoes sorologicas empregadas para diagnostico da doenca de Chagas. Rev bras biol. 1981;41(4):789-95.

66. Avila JL, Rojas M. A galactosyl(alpha 1-3)mannose epitope on phospholipids of Leishmania mexicana and L. braziliensis is recognized by trypanosomatid-infected human sera. J Clin Microbiol. 1990;28(7):1530-7.

67. Gazzinelli RT, Leme VM, Cancado JR, Gazzinelli G, Scharfstein J. Identification and partial characterization of Trypanosoma cruzi antigens recognized by T cells and immune sera from patients with Chagas' disease. Infection and Immunity. 1990;58(5):1437-.

68. Saez-Alquezar A, Salles NA, Sabino EC. Serological diagnosis of Chagas disease in blood bank. Mem Inst Oswaldo Cruz. 1995;90:34-5.

69. Kirchhoff LV, Gam AA, Gusmao RA, Goldsmith RS, Rezende JM, Rassi A. Increased specificity of serodiagnosis of Chagas' disease by detection of antibody to the 72- and 90-kilodalton glycoproteins of Trypanosoma cruzi. J Infect Dis. 1987 Mar;155(3):561-4.

70. Lopes JD, Caulada Z, Barbieri CL, Camargo EP. Cross-reactivity between Trypanosoma cruzi and insect trypanosomatids as a basis for the diagnosos of Chagas' disease. Am J Trop Med Hyg. 1981 Nov;30(6):1183-8.

71. Camargo ME, Segura EL, Kagan IG, Souza JM, Carvalheiro Jda R, Yanovsky JF, et al. Three years of collaboration on the standardization of Chagas' disease serodiagnosis in the Americas: an appraisal. Bull Pan Am Health Organ. 1986;20(3):233-44.

72. Levin MJ, Franco da Silveira J, Frasch ACC, Camargo ME, Lafon S, Degrave WM, et al. Recombinant Trypanosoma cruzi antigens and Chagas' disease diagnosis: analysis of a workshop. FEMS Microbiology Letters. 1991;89(1):11-9.

73. Marcipar A, Barnes S, Lentwojt E, Broun G. Immunoenzymatic determination of antibody-bound soluble antigens of Trypanosoma cruzi. Applied Biochemistry and Biotechnology. 1982;7(6):459-62.

74. Scharfstein J, Luquetti A, Murta ACM, Senna M, Rezende JM, Rassi A, et al. Chagas' Disease: Serodiagnosis with Purified GP25 Antigen. Am J Trop Med Hyg. 1985;34(6):1153-60.

75. Dragon EA, Brothers VM, Wrightsman RA, Manning J. A Mr 90 000 surface polypeptide of Trypanosoma cruzi as a candidate for a Chagas' disease diagnostic antigen. Molecular and Biochemical Parasitology. 1985;16(3):213-29.

76. Grogl M, Kuhn RE. Identification of antigens of culture forms of Trypanosoma cruzi and Trypanosoma rangeli recognized by sera from patients with chronic Chagas' disease. J Parasitol. 1984 Oct;70(5):822-4.

77. Gea S, Evequoz C, Rodriguez P, Vottero-Cima E. Detection of T. cruzi antigens reactive with IgG antibodies from patients with chronic Chagas' disease. Medicina (B Aires). 1986;46(2):163-6.

78. Katzin AM, Colli W. Lectin receptors in Trypanosoma cruzi. An N-acetyl-D-glucosamine-containing surface glycoprotein specific for the trypomastigote stage. Biochim Biophys Acta. 1983 Jan 19;727(2):403-11.

79. Levin MJ, Mesri E, Benarous R, Levitus G, Schijman A, Levy-Yeyati P, et al. Identification of major Trypanosoma cruzi antigenic determinants in chronic Chagas' heart disease. Am J Trop Med Hyg. 1989 Nov;41(5):530-8.

80. Galaz P, Garcia S, Mercado R, Orrego E, Pagliero B, Contreras Mdel C, et al. [Parasitological and epidemiological aspects of Trypanosoma cruzi seropositive blood donors]. Rev Med Chil. 2007 Oct;135(10):1291-5.

81. Furucho CR, Umezawa ES, Almeida I, Freitas VL, Bezerra R, Nunes EV, et al. Inconclusive results in conventional serological screening for Chagas' disease in blood banks: evaluation of cellular and humoral response. Trop Med Int Health. 2008 Dec;13(12):1527-33.

82. Villagrán ME, Sánchez-Moreno M, Marín C, Uribe M, de la Cruz JJ, de Diego JA. Seroprevalence to Trypanosoma cruzi in rural communities of the state of Querétaro (Mexico): statistical evaluation of tests. Clinical Biochemistry. 2009;42(1-2):12-6.

83. Adams ER, Hamilton PB. New molecular tools for the indentification of trypanosome species. Future Microbiology. 2008;3(2):167-76.

84. Houghton RL, Stevens YY, Hjerrild K, Guderian J, Okamoto M, Kabir M, et al. Lateral flow immunoassay for diagnosis of trypanosoma cruzi infection with high correlation to the radioimmunoprecipitation assay. Clinical and Vaccine Immunology. 2009;16(4):515-20.

85. Verani JR, Seitz A, Gilman RH, Lafuente C, Galdos-Cardenas G, Kawai V, et al. Geographie variation in the sensitivity of recombinant antigen-based rapid tests for chronic trypanosoma cruzi infection. American Journal of Tropical Medicine and Hygiene. 2009;80(3):410-5.

86. Roddy P, Goiri J, Flevaud L, Palma PP, Morote S, Lima N, et al. Field evaluation of a rapid immunochromatographic assay for detection of Trypanosoma cruzi infection by use of whole blood. Journal of Clinical Microbiology. 2008;46(6):2022-7.

87. Coronado X, Zulantay I, Albrecht H, Rozas M, Apt W, Ortiz S, et al. Variation in Trypanosoma cruzi clonal composition detected in blood patients and xenodiagnosis triatomines: Implications in the molecular epidemiology of Chile. American Journal of Tropical Medicine and Hygiene. 2006;74(6):1008-12.

88. Duffy T, Bisio M, Altcheh J, Burgos JM, Diez M, Levin MJ, et al. Accurate real-time PCR strategy for monitoring bloodstream parasitic loads in chagas disease patients. PLoS Negl Trop Dis. 2009;3(4):e419.
