# Supplementary material for: ELISA versus PCR for diagnosis of chronic Chagas disease: systematic review and meta-analysis
Source: BMC Infect Dis. 2010 Nov 25;10:337. doi: 10.1186/1471-2334-10-337 (PMC3004908; doi:10.1186/1471-2334-10-337)
Supplement: Additional file 9 — Chart with strategies used on remote databases search. [file 1471-2334-10-337-S9.DOC]

PubMed/Medline in June 6^th^, 2007; updated in April 20^th^, 2009:

*("Chagas Disease"[MeSH] OR "Trypanosoma cruzi"[MeSH]) AND (ELISA OR (enzyme AND linked AND assay) OR PCR OR (polymerase AND chain AND reaction))) AND (sensitiv*[Title/Abstract] OR sensitivity and specificity[MeSH Terms] OR diagnos*[Title/Abstract] OR diagnosis[MeSH:noexp] OR diagnostic *[MeSH:noexp] OR diagnosis,differential[MeSH:noexp] OR diagnosis[Subheading:noexp] OR "Reproducibility of Results"[Mesh] OR reliability OR reproducibility)*

SCOPUS in June 6^th^, 2007; updated in April 20^th^, 2009:

‘*(TITLE-ABS-KEY("Chagas Disease") OR TITLE-ABS-KEY("Trypanosoma cruzi")) AND (ALL(ELISA) OR (ALL(enzyme) AND ALL(linked) AND ALL(assay)) OR PCR OR (ALL(polymerase) AND ALL(chain) AND ALL(reaction))) AND (TITLE-ABS-KEY(sensitiv*) OR KEY("sensitivity and specificity") OR TITLE-ABS-KEY(diagnos*) OR KEY(diagnosis) OR KEY(diagnostic*) OR KEY("diagnosis,differential") OR KEY("differential diagnosis") OR TITLE-ABS-KEY(reliability) OR TITLE-ABS-KEY(reproducibility) OR KEY("Reproducibility of Results"))’*

ISIWeb/Web of Science in June 6^th^, 2007; updated in April 20^th^, 2009:

‘*(("Chagas Disease" OR "Trypanosoma cruzi") AND (ELISA OR (enzyme AND linked AND assay) OR PCR OR (polymerase AND chain AND reaction)) AND (sensitiv* OR "sensitivity and specificity" OR diagnos* OR diagnostic* OR "diagnosis,differential" OR "differential diagnosis" OR reliability OR reproducibility))*’

LILACS in June 6^th^, 2007; updated in April 20^th^, 2009:

*‘((([MH] ("doença de chagas")) or ([MH]"TRYPANOSOMA CRUZI")) and (((elisa or (enzyme and linked and assay) or pcr or (polymerase and chain and reaction)))) and (sensib$ or ([MH]"SENSIBILIDADE E ESPECIFICIDADE") or ([MH]"DIAGNOSTICO DIFERENCIAL") or ([MH]"DIAGNOSTICO") or diagnos$ ))’*

EMBASE/Ovid in June 6^th^, 2007:

*‘(Chagas disease.sh or Trypanosoma cruzi.sh.) and (ELISA.af or (enzime.af and linked.af and assay.af) or PCR.af or (polymerase.af and chain.af and reaction.af) and (sensitiv$.ab,ti or sensitivity and specificity.sh or diagnos$.ab,ti or (diagnosis or diagnostic acuracy).sh or (diagnostic error or diagnostic approach route).sh. or differential diagnosis.sh. or diagnosis.fs. or reliability.sh. or reliability. or reproducibility.af.)’*

EMBASE website in April 20^th^, 2009:

*‘('reproducibility'/syn OR 'reproducibility'/exp OR 'reproducibility') OR ('reliability'/exp OR 'reliability' OR 'reliability'/syn) OR ('differential diagnosis'/exp OR 'differential diagnosis') OR ('diagnostic approach route'/exp OR 'diagnostic approach route') OR ('diagnostic error'/exp OR 'diagnostic error') OR ('diagnostic accuracy'/exp OR 'diagnostic accuracy') OR ('diagnosis'/exp OR 'diagnosis' OR 'diagnos') OR ('sensitiv' OR 'sensitivity and specificity'/exp/mj) AND (('trypanosoma cruzi'/exp/mj OR 'trypanosoma cruzi'/de) OR ('chagas disease'/exp/mj OR 'chagas disease'/de)) AND ((('elisa'/exp OR 'elisa'/de) OR ('enzyme linked immunosorbent assay'/exp/mj OR 'enzyme linked immunosorbent assay'/de)) OR ('pcr' OR ('polymerase chain reaction'/exp/mj OR 'polymerase chain reaction'/de)))’*
